# Crystal structure and Hirshfeld surface analysis of 1,3,3-trimethyl-2,6-di­phenyl­piperidin-4-yl 2-phenyl­prop-2-enoate

**DOI:** 10.1107/S2056989025008709

**Published:** 2025-10-09

**Authors:** Aranganathan Ananthabharathi, Sekar Janarthanan, Mannathusamy Gopalakrishnan, Srinivasan Pazhamalai, Sivashanmugam Selvanayagam

**Affiliations:** a Department of Chemistry Annamalai University, Annamalainagar Chidambaram 608 002 India; b PG & Research Department of Physics, Government Arts College, Melur 625 106, India; Vienna University of Technology, Austria

**Keywords:** piperidine derivative, inter­molecular hydrogen bonds, crystal structure

## Abstract

The title compound, C_29_H_31_NO_2_, is a multi-substituted piperidine derivative in which the piperidine ring adopts a chair conformation. Inter­molecular C—H⋯O hydrogen bonds as well as C—H⋯π inter­actions are observed in the crystal, leading to the formation of inversion dimers and chains, respectively. The inter­molecular inter­actions were qu­anti­fied and analysed using Hirshfeld surface analysis, revealing that H⋯H inter­actions contribute the most (70.5%) to the crystal packing.

## Abstract 2

The title compound is a multi-substituted piperidine derivative in which the piperidine ring adopts a chair conformation.

## 1. Chemical context

Compounds with piperidine-based scaffolds represent an important class of nitro­gen heterocycles, occurring widely in natural alkaloids and serving as versatile building blocks in medicinal chemistry due to their wide-ranging pharmacological importance. Examples are arecoline and pethidine. Moreover, piperidine derivatives show therapeutic properties as anti-cancer, anti­microbial, analgesic, anti-inflammatory, or anti­psychotic agents (Abdelshaheed *et al.*, 2021 ▸). Structural modifications on the piperidine framework often modulate biological activity, lipophilicity, and supra­molecular inter­actions, making them valuable targets for both medicinal and crystallographic studies (Mitra *et al.*, 2022 ▸; Grover *et al.*, 2023 ▸).
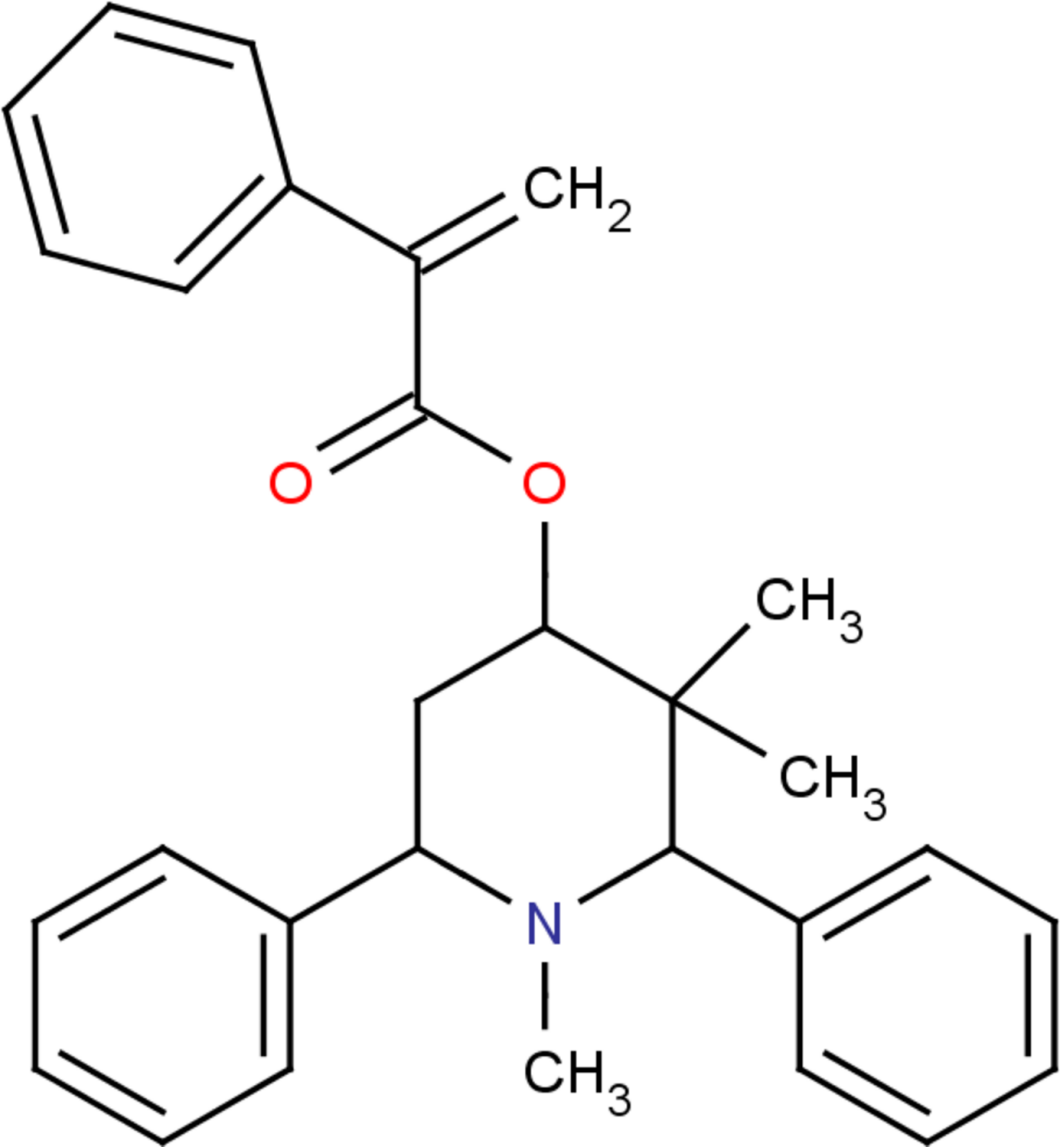


In the context of piperidine frameworks given above, we synthesized the title compound, (I), C_29_H_31_NO_2_, and report here its mol­ecular and crystal structures, as well as the results of a Hirshfeld surface analysis.

## 2. Structural commentary

**Figure 1:**
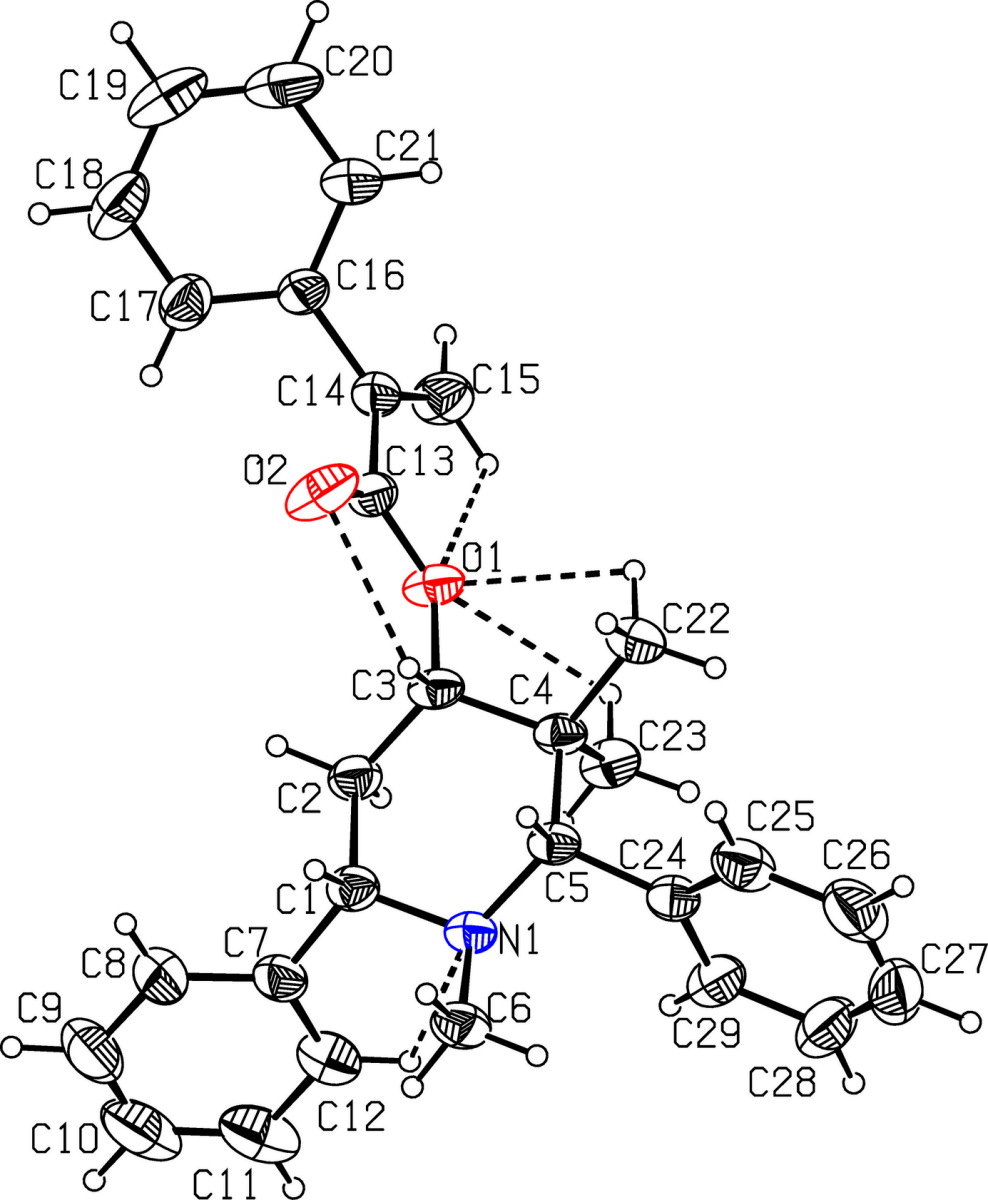
The mol­ecular structure of compound (I), showing the atom labelling. Displacement ellipsoids are drawn at the 30% probability level. Intra­molecular short contacts are shown as dashed lines.

The mol­ecular structure of (I) is shown in Fig. 1 ▸. The C13=O2 [1.198 (2) Å] and C14=C15 [1.319 (2) Å] bond lengths confirm the double-bond character. The piperidine ring (N1/C1–C5) adopts a chair conformation with puckering parameters (Cremer & Pople, 1975 ▸) of *q*_2_ = 0.101 (2) Å, *q*_3_ = −0.553 (2) Å, *Q*_T_ = 0.562 (2) Å, θ = 169.7 (2)° and φ = 24.4 (9)°. Atoms C1 and C4 deviate by 0.574 (2) and −0.726 (1) Å, respectively, from the least-squares plane through the remaining four atoms (N1/C2/C3/C5) of the ring. The mean plane calculation of the prop-2-enoic acid moiety (O1/C13/O2/C14/C15) reveals that atoms C15 and C14 deviate by −0.050 (2) and 0.038 (2) Å, respectively, from the plane. This moiety makes a dihedral angle of 54.1 (1)° with respect to the attached phenyl ring (C16–C21). The two other phenyl rings (C7–C12 and C24–C29) subtend a dihedral angle of 26.6 (1)°.

## 3. Supra­molecular features

**Figure 2:**
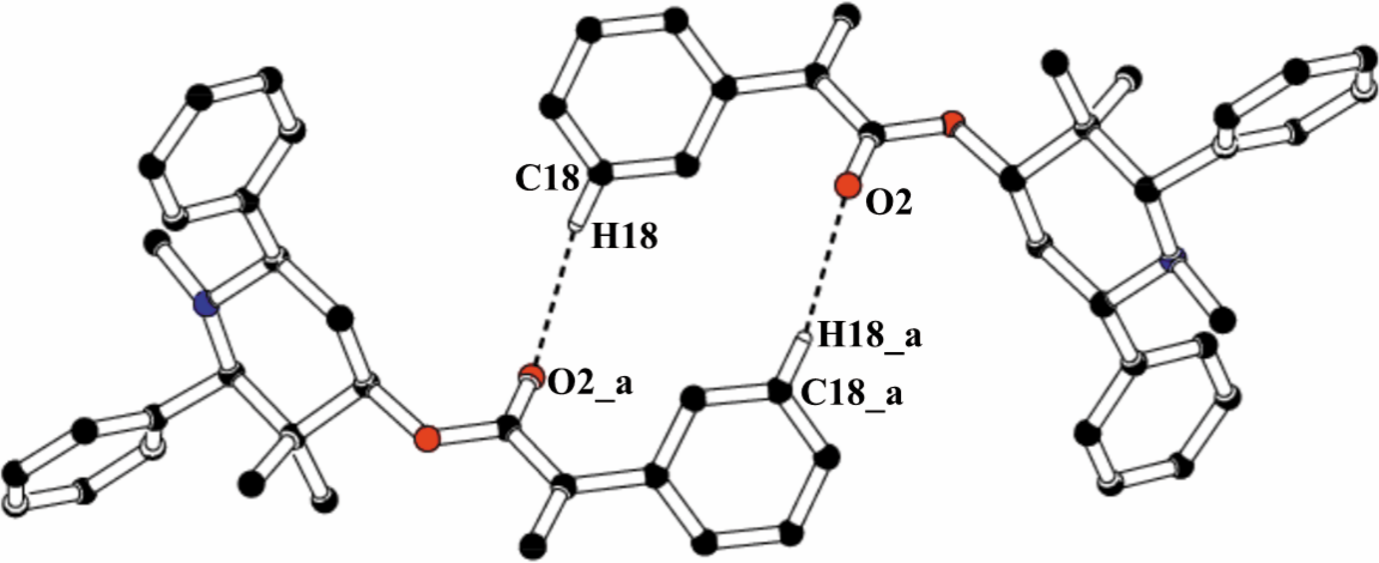
The formation of an inversion dimer through C—H⋯O hydrogen bonds in the crystal structure of (I). [Symmetry code: (*a*) −*x*, −*y* − 1, −*z*.]

**Figure 3:**
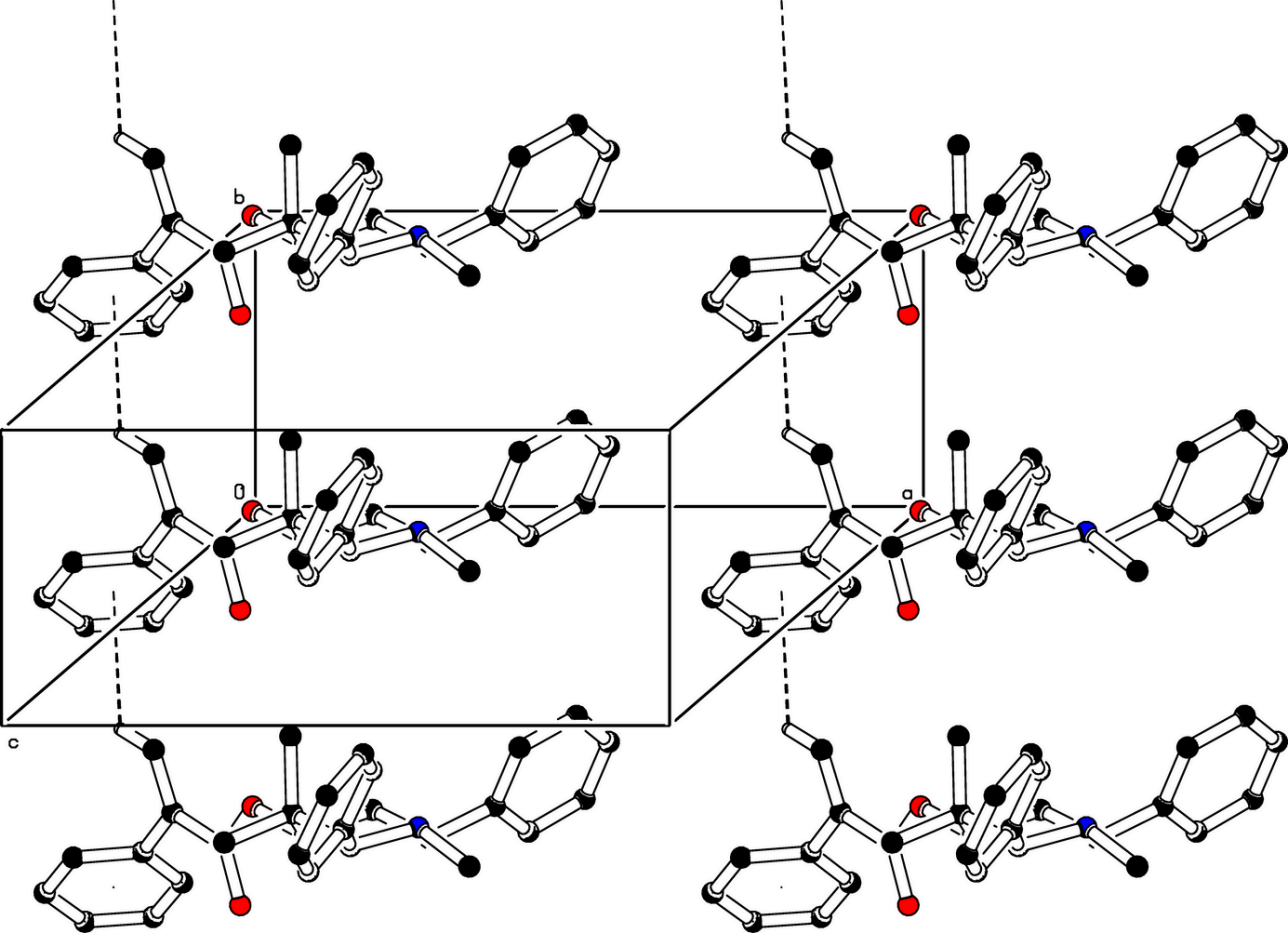
The crystal packing of (I). Inter­molecular C—H⋯π inter­actions are shown as dashed lines. For clarity, H atoms not involved in these inter­actions have been omitted.

In the crystal, mol­ecules associate pairwise *via* C18—H18⋯O2^i^ hydrogen bonds (Table 1 ▸) into inversion dimers with an 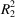
(14) graph-set motif (Etter *et al.*, 1990 ▸; Bernstein *et al.*, 1995 ▸), as shown in Fig. 2 ▸. In addition, mol­ecules are linked into a *C*(5) chain motif by C—H⋯π inter­actions, C15—H15*A*⋯*Cg*, where *Cg* is the centroid of the C16–C21 benzene ring of the symmetry-related mol­ecules at *x*, *y* + 1, *z* (Table 1 ▸). These *C*(5) chains run in a parallel manner along the [010] direction (Fig. 3 ▸).

## 4. Hirshfeld surface analysis

**Figure 4:**
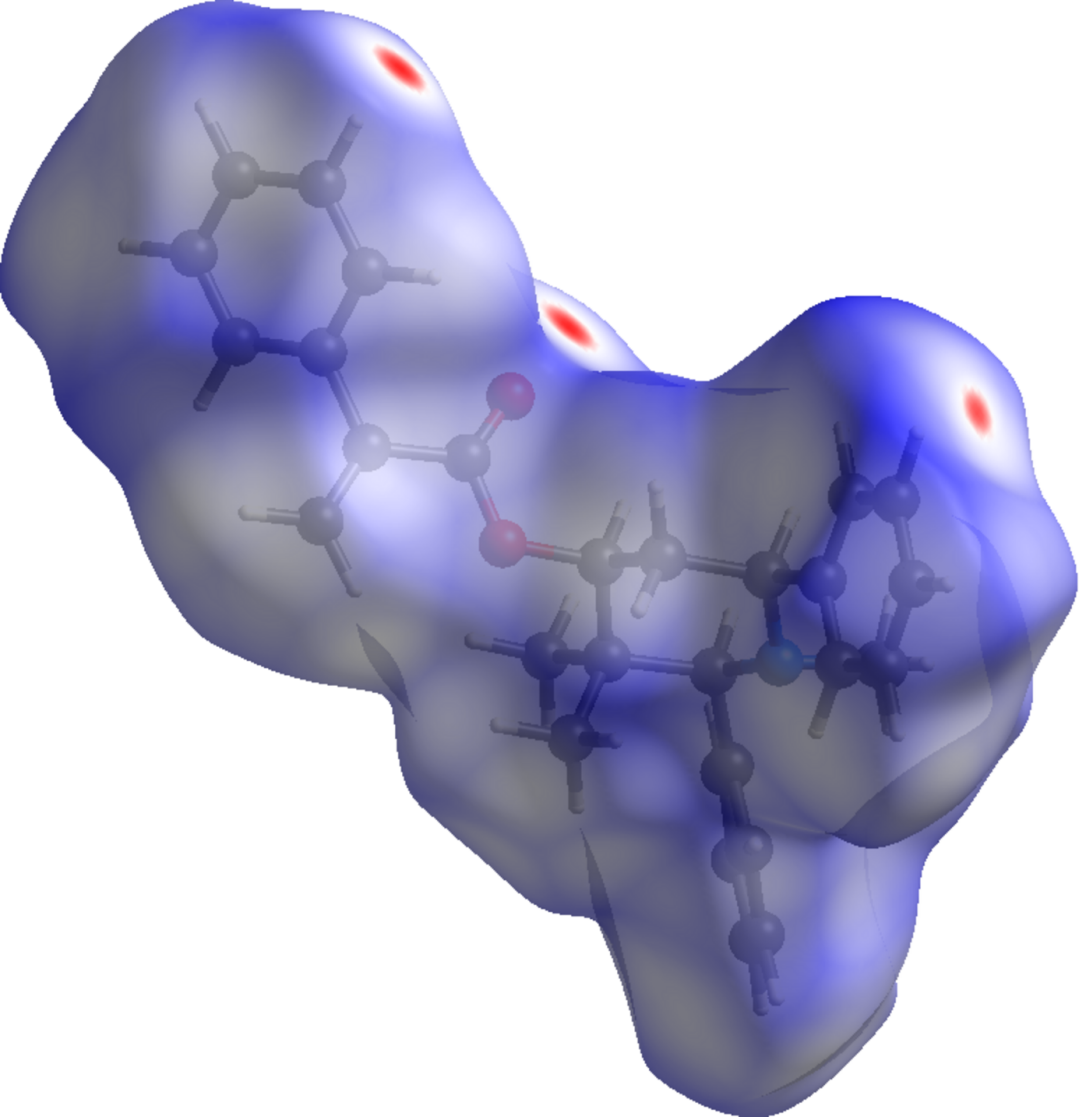
A view of the Hirshfeld surface mapped over *d*_norm_ for compound (I).

In order to further characterize and qu­antify the inter­molecular inter­actions in the title compound, a Hirshfeld surface (HS) analysis (Spackman & Jayatilaka, 2009 ▸) was carried out using *CrystalExplorer* (Spackman *et al.*, 2021 ▸). The HS mapped over *d*_norm_ is illustrated in Fig. 4 ▸ where the deep-red spots at O2 and H18 are indicative of the inter­molecular C—H⋯O hydrogen bonds discussed in the previous section.

**Figure 5:**
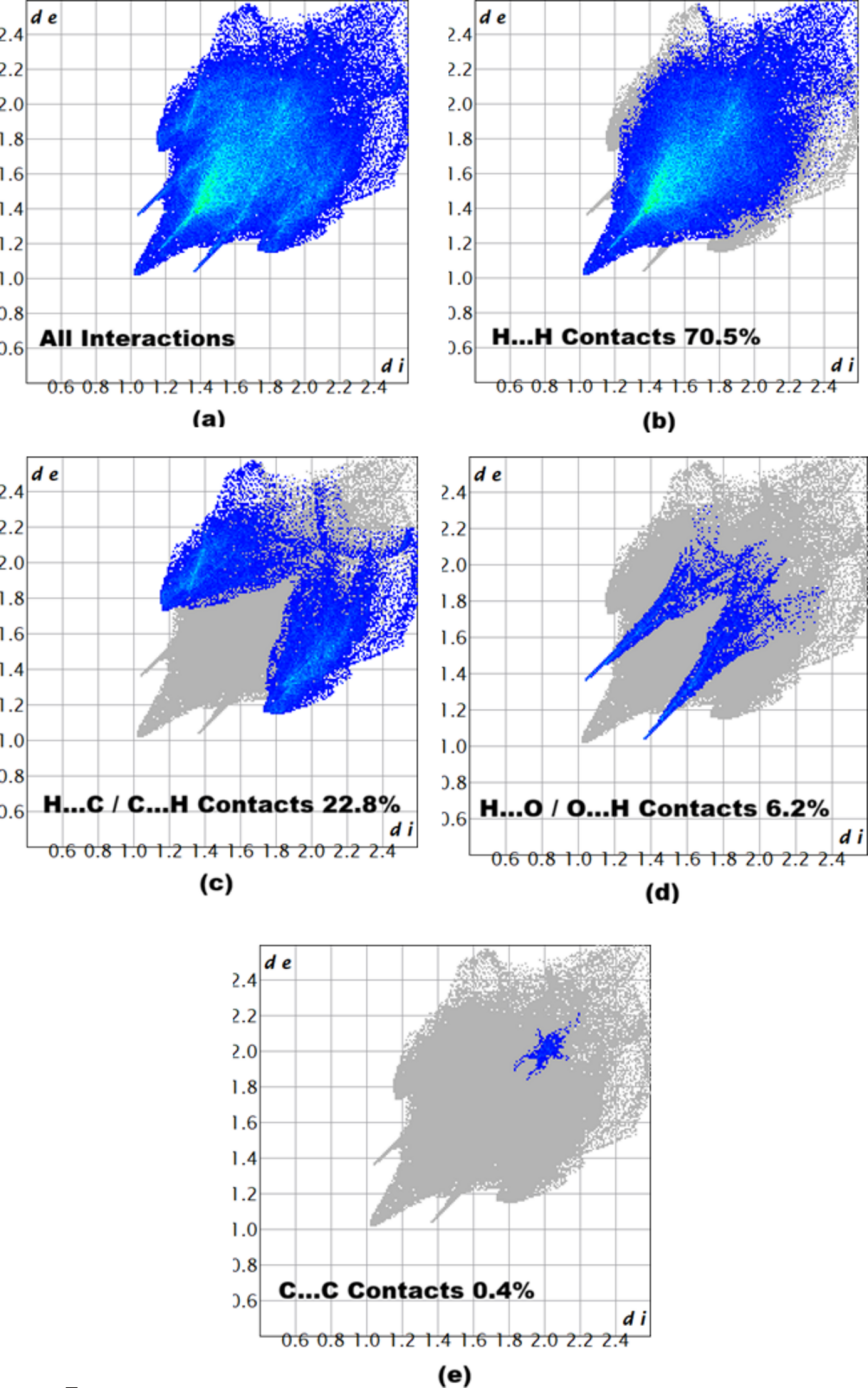
Two-dimensional fingerprint plots for compound (I), showing all inter­actions, and delineated into H⋯H, H⋯C/C⋯H, H⋯O/O⋯H and C⋯C inter­actions. The *d*_i_ and *d*_e_ values are the closest inter­nal and external distances (in Å) from given points on the Hirshfeld surface.

The associated two-dimensional fingerprint plots (McKinnon *et al.*, 2007 ▸) provide qu­anti­tative information about the non-covalent inter­actions in the crystal packing in terms of the percentage contribution of the inter­atomic contacts (Spackman & McKinnon, 2002 ▸). As shown in Fig. 5 ▸, the overall two-dimensional fingerprint plot for compound (I) is delineated in H⋯H, H⋯C/C⋯H, H⋯O/O⋯H and C⋯C contacts, revealing that H⋯H and H⋯C/C⋯H are the main contributors to the crystal packing.

## 5. Synthesis and crystallization

>A solution of 1,3,3-trimethyl-2,6-di­phenyl­piperidin-4-ol (0.5 g), tropic acid (0.29 g), *N*,*N*′-di­cyclo­hexyl­carbodi­imide (0.74 g) and *N*,*N*-di­methyl­amino­pyridine 0.25 g) in dry di­chloro­methane (30 ml) was refluxed at 313 K for 6–8 h. After filtration, the organic layer was washed with aqueous NaHCO_3_ (10%_wt_) and brine. The combined organic layer was then concentrated under reduced pressure. The crude ester was purified by column chromatography (silica gel 100–200 mesh, petroleum ether/ethyl acetate *v*:*v* 9:1) and recrystallized from aceto­nitrile solution (99%), affording colourless crystals of the title compound [see Jordan *et al.*, 2021 ▸) for the synthesis procedure for esterification by using DCC and DMAP catalysts].

## 6. Refinement

Crystal data, data collection and structure refinement details are summarized in Table 2 ▸. H atoms were placed in idealized positions and allowed to ride on their parent atoms: C—H = 0.93–0.98 Å, with *U*_iso_(H) = 1.5*U*_eq_(C-meth­yl) and 1.2*U*_eq_ for other H atoms.

## Supplementary Material

Crystal structure: contains datablock(s) I, global. DOI: 10.1107/S2056989025008709/wm5771sup1.cif

Structure factors: contains datablock(s) I. DOI: 10.1107/S2056989025008709/wm5771Isup2.hkl

Supporting information file. DOI: 10.1107/S2056989025008709/wm5771Isup3.cml

CCDC reference: 2493059

Additional supporting information:  crystallographic information; 3D view; checkCIF report

## Figures and Tables

**Table 1:** Hydrogen-bond geometry (Å, °) *Cg* is the centroid of the C16–C21 phenyl ring.

*D*—H⋯*A*	*D*—H	H⋯*A*	*D*⋯*A*	*D*—H⋯*A*
C18—H18⋯O2^i^	0.93	2.55	3.473 (3)	170
C15—H15*A*⋯*Cg*^ii^	0.93	2.94	3.613 (2)	130

Symmetry codes: (i) 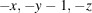; (ii) 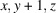.

**Table 2:** Experimental details

Crystal data	
Chemical formula	C_29_H_31_NO_2_
*M*_r_	425.55
Crystal system, space group	Monoclinic, *P*2_1_/*c*
Temperature (K)	300
*a*, *b*, *c* (Å)	13.2165 (8), 5.8983 (4), 31.503 (2)
β (°)	99.174 (2)
*V* (Å^3^)	2424.4 (3)
*Z*	4
Radiation type	Mo *K*α
μ (mm^−1^)	0.07
Crystal size (mm)	0.36 × 0.22 × 0.16

Data collection	
Diffractometer	Bruker APEXII CCD
Absorption correction	Multi-scan (*SADABS*; Krause *et al.*, 2015 ▸)
*T*_min_, *T*_max_	0.975, 0.988
No. of measured, independent and observed [*I* > 2σ(*I*)] reflections	43823, 6019, 4053
*R*_int_	0.041
(sin θ/λ)_max_ (Å^−1^)	0.667

Refinement	
*R*[*F*^2^ > 2σ(*F*^2^)], *wR*(*F*^2^), *S*	0.052, 0.168, 1.03
No. of reflections	6019
No. of parameters	290
H-atom treatment	H-atom parameters constrained
Δρ_max_, Δρ_min_ (e Å^−3^)	0.18, −0.20

Computer programs: *APEX3* and *SAINT* (Bruker, 2017 ▸), *SHELXT* (Sheldrick, 2015*a* ▸), *SHELXL* (Sheldrick, 2015*b* ▸), *ORTEP-3 for Windows* (Farrugia, 2012 ▸) and *PLATON* (Spek, 2020 ▸).

## supplementary crystallographic information

### 1,3,3-Trimethyl-2,6-diphenylpiperidin-4-yl 2-phenylprop-2-enoate Crystal data

**Table:**

C_29_H_31_NO_2_	*F*(000) = 912
*M**_r_* = 425.55	*D*_x_ = 1.166 Mg m^−^^3^
Monoclinic, *P*2_1_/*c*	Mo *K*α radiation, λ = 0.71073 Å
*a* = 13.2165 (8) Å	Cell parameters from 9887 reflections
*b* = 5.8983 (4) Å	θ = 2.8–27.9°
*c* = 31.503 (2) Å	µ = 0.07 mm^−^^1^
β = 99.174 (2)°	*T* = 300 K
*V* = 2424.4 (3) Å^3^	Block, colourless
*Z* = 4	0.36 × 0.22 × 0.16 mm

### 1,3,3-Trimethyl-2,6-diphenylpiperidin-4-yl 2-phenylprop-2-enoate Data collection

**Table:**

Bruker APEXII CCD diffractometer	4053 reflections with *I* > 2σ(*I*)
Radiation source: i-mu-s microfocus source	*R*_int_ = 0.041
ω and φ scans	θ_max_ = 28.3°, θ_min_ = 2.2°
Absorption correction: multi-scan (SADABS; Krause *et al.*, 2015)	*h* = −17→17
*T*_min_ = 0.975, *T*_max_ = 0.988	*k* = −7→7
43823 measured reflections	*l* = −41→42
6019 independent reflections	

### 1,3,3-Trimethyl-2,6-diphenylpiperidin-4-yl 2-phenylprop-2-enoate Refinement

**Table:**

Refinement on *F*^2^	Hydrogen site location: inferred from neighbouring sites
Least-squares matrix: full	H-atom parameters constrained
*R*[*F*^2^ > 2σ(*F*^2^)] = 0.052	*w* = 1/[σ^2^(*F*_o_^2^) + (0.0655*P*)^2^ + 0.7185*P*] where *P* = (*F*_o_^2^ + 2*F*_c_^2^)/3
*wR*(*F*^2^) = 0.168	(Δ/σ)_max_ < 0.001
*S* = 1.03	Δρ_max_ = 0.18 e Å^−^^3^
6019 reflections	Δρ_min_ = −0.20 e Å^−^^3^
290 parameters	Extinction correction: SHELXL (Sheldrick 2015b), Fc^*^=kFc[1+0.001xFc^2^λ^3^/sin(2θ)]^-1/4^
0 restraints	Extinction coefficient: 0.0181 (18)

### 1,3,3-Trimethyl-2,6-diphenylpiperidin-4-yl 2-phenylprop-2-enoate Special details

**Table:**

Geometry. All esds (except the esd in the dihedral angle between two l.s. planes) are estimated using the full covariance matrix. The cell esds are taken into account individually in the estimation of esds in distances, angles and torsion angles; correlations between esds in cell parameters are only used when they are defined by crystal symmetry. An approximate (isotropic) treatment of cell esds is used for estimating esds involving l.s. planes.

### 1,3,3-Trimethyl-2,6-diphenylpiperidin-4-yl 2-phenylprop-2-enoate Fractional atomic coordinates and isotropic or equivalent isotropic displacement parameters (Å^2^)

**Table:**

	*x*	*y*	*z*	*U*_iso_*/*U*_eq_	
O1	0.03926 (8)	0.06917 (18)	0.11420 (4)	0.0611 (3)	
O2	0.01154 (12)	−0.2824 (2)	0.08983 (5)	0.0893 (5)	
N1	0.32017 (9)	0.0625 (2)	0.20033 (4)	0.0513 (3)	
C1	0.32010 (12)	−0.0081 (3)	0.15561 (5)	0.0552 (4)	
H1	0.324364	−0.173859	0.154789	0.066*	
C2	0.22312 (12)	0.0663 (3)	0.12589 (5)	0.0588 (4)	
H2A	0.223440	0.229827	0.122584	0.071*	
H2B	0.221726	−0.001316	0.097742	0.071*	
C3	0.12920 (11)	−0.0044 (3)	0.14390 (5)	0.0522 (4)	
H3	0.128246	−0.169932	0.146411	0.063*	
C4	0.12649 (11)	0.1003 (2)	0.18778 (5)	0.0496 (4)	
C5	0.22462 (10)	0.0116 (2)	0.21721 (5)	0.0485 (3)	
H5	0.218782	−0.153685	0.218778	0.058*	
C6	0.40668 (12)	−0.0499 (3)	0.22744 (6)	0.0677 (5)	
H6A	0.408156	−0.006072	0.256875	0.101*	
H6B	0.398667	−0.211382	0.224883	0.101*	
H6C	0.469652	−0.005757	0.218237	0.101*	
C7	0.41087 (12)	0.0892 (3)	0.13812 (6)	0.0631 (4)	
C8	0.44879 (16)	−0.0219 (5)	0.10551 (7)	0.0933 (7)	
H8	0.422477	−0.162846	0.096242	0.112*	
C9	0.5256 (2)	0.0747 (8)	0.08650 (9)	0.1305 (13)	
H9	0.549227	−0.000456	0.064059	0.157*	
C10	0.5670 (2)	0.2776 (8)	0.10007 (11)	0.1260 (13)	
H10	0.619400	0.339895	0.087301	0.151*	
C11	0.53136 (18)	0.3897 (5)	0.13259 (10)	0.1038 (8)	
H11	0.559606	0.528631	0.142098	0.125*	
C12	0.45310 (15)	0.2967 (4)	0.15145 (7)	0.0782 (6)	
H12	0.428654	0.374833	0.173367	0.094*	
C13	−0.01149 (11)	−0.0858 (3)	0.08867 (5)	0.0518 (4)	
C14	−0.10124 (11)	0.0090 (3)	0.05961 (5)	0.0525 (4)	
C15	−0.12826 (15)	0.2228 (3)	0.06286 (6)	0.0742 (5)	
H15A	−0.185104	0.279718	0.044785	0.089*	
H15B	−0.090481	0.316592	0.083223	0.089*	
C16	−0.15837 (12)	−0.1489 (3)	0.02779 (5)	0.0564 (4)	
C17	−0.10977 (16)	−0.2729 (4)	−0.00014 (5)	0.0708 (5)	
H17	−0.039023	−0.262514	0.001575	0.085*	
C18	−0.1648 (2)	−0.4116 (4)	−0.03050 (7)	0.0958 (8)	
H18	−0.130994	−0.493464	−0.049229	0.115*	
C19	−0.2677 (3)	−0.4298 (5)	−0.03334 (8)	0.1134 (10)	
H19	−0.304484	−0.523105	−0.054083	0.136*	
C20	−0.3175 (2)	−0.3114 (6)	−0.00582 (9)	0.1157 (10)	
H20	−0.388066	−0.325807	−0.007462	0.139*	
C21	−0.26323 (14)	−0.1701 (5)	0.02451 (7)	0.0876 (7)	
H21	−0.297792	−0.088081	0.042958	0.105*	
C22	0.03109 (12)	0.0142 (3)	0.20437 (6)	0.0672 (5)	
H22A	0.028268	0.078936	0.232114	0.101*	
H22B	−0.028940	0.057586	0.184710	0.101*	
H22C	0.033936	−0.148016	0.206701	0.101*	
C23	0.12310 (14)	0.3598 (3)	0.18558 (6)	0.0634 (4)	
H23A	0.121449	0.420168	0.213756	0.095*	
H23B	0.182901	0.414792	0.175117	0.095*	
H23C	0.062811	0.406964	0.166470	0.095*	
C24	0.23188 (12)	0.1029 (3)	0.26259 (5)	0.0561 (4)	
C25	0.19071 (14)	−0.0174 (4)	0.29329 (6)	0.0799 (6)	
H25	0.161466	−0.158856	0.286507	0.096*	
C26	0.19262 (19)	0.0714 (7)	0.33434 (8)	0.1145 (11)	
H26	0.162999	−0.008895	0.354612	0.137*	
C27	0.2381 (2)	0.2771 (8)	0.34496 (9)	0.1189 (12)	
H27	0.239505	0.335912	0.372438	0.143*	
C28	0.2812 (2)	0.3951 (5)	0.31534 (8)	0.0972 (8)	
H28	0.312974	0.533335	0.322796	0.117*	
C29	0.27811 (15)	0.3109 (3)	0.27429 (6)	0.0705 (5)	
H29	0.307183	0.393851	0.254201	0.085*	

### 1,3,3-Trimethyl-2,6-diphenylpiperidin-4-yl 2-phenylprop-2-enoate Atomic displacement parameters (Å^2^)

**Table:**

	*U*^11^	*U*^22^	*U*^33^	*U*^12^	*U*^13^	*U*^23^
O1	0.0508 (6)	0.0468 (6)	0.0766 (7)	0.0009 (5)	−0.0178 (5)	−0.0037 (5)
O2	0.1007 (10)	0.0557 (8)	0.0928 (10)	0.0142 (7)	−0.0415 (8)	−0.0174 (7)
N1	0.0407 (6)	0.0533 (7)	0.0568 (7)	−0.0003 (5)	−0.0017 (5)	0.0011 (6)
C1	0.0486 (8)	0.0503 (9)	0.0638 (9)	0.0032 (7)	0.0002 (7)	−0.0031 (7)
C2	0.0496 (8)	0.0642 (10)	0.0588 (9)	0.0016 (7)	−0.0033 (7)	−0.0036 (8)
C3	0.0428 (7)	0.0413 (8)	0.0664 (9)	0.0017 (6)	−0.0098 (6)	−0.0019 (7)
C4	0.0417 (7)	0.0390 (7)	0.0645 (9)	−0.0008 (6)	−0.0020 (6)	−0.0011 (6)
C5	0.0431 (7)	0.0375 (7)	0.0622 (8)	−0.0021 (6)	0.0000 (6)	0.0019 (6)
C6	0.0457 (8)	0.0804 (12)	0.0723 (11)	0.0086 (8)	−0.0046 (7)	0.0084 (9)
C7	0.0442 (8)	0.0781 (12)	0.0647 (10)	0.0083 (8)	0.0020 (7)	0.0031 (9)
C8	0.0619 (11)	0.133 (2)	0.0858 (14)	0.0136 (13)	0.0135 (10)	−0.0206 (14)
C9	0.0649 (15)	0.236 (4)	0.0952 (18)	0.021 (2)	0.0280 (13)	−0.007 (2)
C10	0.0565 (14)	0.212 (4)	0.111 (2)	0.0020 (19)	0.0203 (14)	0.056 (2)
C11	0.0638 (13)	0.116 (2)	0.131 (2)	−0.0098 (13)	0.0130 (14)	0.0376 (17)
C12	0.0596 (10)	0.0799 (14)	0.0959 (14)	−0.0054 (10)	0.0141 (10)	0.0095 (11)
C13	0.0477 (8)	0.0516 (9)	0.0535 (8)	−0.0001 (7)	−0.0004 (6)	−0.0047 (7)
C14	0.0441 (7)	0.0599 (9)	0.0520 (8)	0.0031 (7)	0.0030 (6)	0.0002 (7)
C15	0.0654 (11)	0.0712 (12)	0.0792 (12)	0.0136 (9)	−0.0098 (9)	−0.0016 (10)
C16	0.0502 (8)	0.0694 (10)	0.0462 (8)	0.0009 (7)	−0.0027 (6)	0.0016 (7)
C17	0.0755 (12)	0.0803 (13)	0.0535 (9)	0.0080 (10)	0.0012 (8)	−0.0048 (9)
C18	0.126 (2)	0.0941 (16)	0.0582 (11)	0.0157 (15)	−0.0134 (12)	−0.0170 (11)
C19	0.130 (2)	0.113 (2)	0.0787 (15)	−0.0208 (18)	−0.0422 (15)	−0.0145 (14)
C20	0.0711 (14)	0.157 (3)	0.1056 (19)	−0.0240 (16)	−0.0276 (13)	−0.0177 (19)
C21	0.0515 (10)	0.1270 (19)	0.0786 (12)	−0.0030 (11)	−0.0071 (9)	−0.0169 (12)
C22	0.0451 (8)	0.0701 (11)	0.0842 (12)	−0.0022 (8)	0.0041 (8)	0.0017 (9)
C23	0.0632 (10)	0.0438 (9)	0.0768 (11)	0.0056 (7)	−0.0087 (8)	−0.0018 (8)
C24	0.0465 (8)	0.0604 (10)	0.0587 (9)	0.0047 (7)	0.0001 (6)	0.0037 (7)
C25	0.0568 (10)	0.1088 (17)	0.0729 (12)	0.0005 (10)	0.0063 (9)	0.0203 (12)
C26	0.0678 (14)	0.208 (4)	0.0691 (14)	0.0195 (19)	0.0144 (11)	0.0227 (18)
C27	0.0841 (17)	0.198 (4)	0.0686 (15)	0.051 (2)	−0.0054 (13)	−0.030 (2)
C28	0.0943 (16)	0.1037 (18)	0.0817 (15)	0.0283 (14)	−0.0223 (13)	−0.0331 (13)
C29	0.0712 (11)	0.0642 (11)	0.0700 (11)	0.0039 (9)	−0.0075 (9)	−0.0096 (9)

### 1,3,3-Trimethyl-2,6-diphenylpiperidin-4-yl 2-phenylprop-2-enoate Geometric parameters (Å, º)

**Table:**

O1—C13	1.3268 (18)	C13—C14	1.487 (2)
O1—C3	1.4566 (17)	C14—C15	1.319 (2)
O2—C13	1.198 (2)	C14—C16	1.484 (2)
N1—C1	1.469 (2)	C15—H15A	0.9300
N1—C6	1.471 (2)	C15—H15B	0.9300
N1—C5	1.4768 (19)	C16—C21	1.379 (2)
C1—C7	1.511 (2)	C16—C17	1.379 (2)
C1—C2	1.526 (2)	C17—C18	1.375 (3)
C1—H1	0.9800	C17—H17	0.9300
C2—C3	1.504 (2)	C18—C19	1.354 (4)
C2—H2A	0.9700	C18—H18	0.9300
C2—H2B	0.9700	C19—C20	1.361 (4)
C3—C4	1.520 (2)	C19—H19	0.9300
C3—H3	0.9800	C20—C21	1.380 (3)
C4—C22	1.527 (2)	C20—H20	0.9300
C4—C23	1.532 (2)	C21—H21	0.9300
C4—C5	1.5593 (19)	C22—H22A	0.9600
C5—C24	1.516 (2)	C22—H22B	0.9600
C5—H5	0.9800	C22—H22C	0.9600
C6—H6A	0.9600	C23—H23A	0.9600
C6—H6B	0.9600	C23—H23B	0.9600
C6—H6C	0.9600	C23—H23C	0.9600
C7—C8	1.378 (3)	C24—C25	1.379 (3)
C7—C12	1.382 (3)	C24—C29	1.394 (2)
C8—C9	1.381 (4)	C25—C26	1.392 (4)
C8—H8	0.9300	C25—H25	0.9300
C9—C10	1.357 (5)	C26—C27	1.372 (5)
C9—H9	0.9300	C26—H26	0.9300
C10—C11	1.365 (5)	C27—C28	1.360 (4)
C10—H10	0.9300	C27—H27	0.9300
C11—C12	1.386 (3)	C28—C29	1.380 (3)
C11—H11	0.9300	C28—H28	0.9300
C12—H12	0.9300	C29—H29	0.9300
			
C13—O1—C3	117.78 (12)	O2—C13—O1	123.16 (14)
C1—N1—C6	108.09 (13)	O2—C13—C14	123.83 (14)
C1—N1—C5	114.69 (11)	O1—C13—C14	112.98 (13)
C6—N1—C5	109.24 (12)	C15—C14—C16	122.53 (15)
N1—C1—C7	111.29 (13)	C15—C14—C13	120.74 (15)
N1—C1—C2	112.27 (13)	C16—C14—C13	116.73 (14)
C7—C1—C2	107.75 (14)	C14—C15—H15A	120.0
N1—C1—H1	108.5	C14—C15—H15B	120.0
C7—C1—H1	108.5	H15A—C15—H15B	120.0
C2—C1—H1	108.5	C21—C16—C17	117.98 (17)
C3—C2—C1	110.60 (14)	C21—C16—C14	120.31 (16)
C3—C2—H2A	109.5	C17—C16—C14	121.68 (15)
C1—C2—H2A	109.5	C18—C17—C16	120.7 (2)
C3—C2—H2B	109.5	C18—C17—H17	119.6
C1—C2—H2B	109.5	C16—C17—H17	119.6
H2A—C2—H2B	108.1	C19—C18—C17	120.5 (2)
O1—C3—C2	108.26 (13)	C19—C18—H18	119.8
O1—C3—C4	109.16 (12)	C17—C18—H18	119.8
C2—C3—C4	111.78 (12)	C18—C19—C20	120.0 (2)
O1—C3—H3	109.2	C18—C19—H19	120.0
C2—C3—H3	109.2	C20—C19—H19	120.0
C4—C3—H3	109.2	C19—C20—C21	120.0 (2)
C3—C4—C22	108.46 (13)	C19—C20—H20	120.0
C3—C4—C23	111.67 (14)	C21—C20—H20	120.0
C22—C4—C23	109.15 (14)	C16—C21—C20	120.8 (2)
C3—C4—C5	105.45 (12)	C16—C21—H21	119.6
C22—C4—C5	109.82 (13)	C20—C21—H21	119.6
C23—C4—C5	112.19 (12)	C4—C22—H22A	109.5
N1—C5—C24	109.76 (12)	C4—C22—H22B	109.5
N1—C5—C4	113.32 (12)	H22A—C22—H22B	109.5
C24—C5—C4	111.28 (12)	C4—C22—H22C	109.5
N1—C5—H5	107.4	H22A—C22—H22C	109.5
C24—C5—H5	107.4	H22B—C22—H22C	109.5
C4—C5—H5	107.4	C4—C23—H23A	109.5
N1—C6—H6A	109.5	C4—C23—H23B	109.5
N1—C6—H6B	109.5	H23A—C23—H23B	109.5
H6A—C6—H6B	109.5	C4—C23—H23C	109.5
N1—C6—H6C	109.5	H23A—C23—H23C	109.5
H6A—C6—H6C	109.5	H23B—C23—H23C	109.5
H6B—C6—H6C	109.5	C25—C24—C29	118.22 (18)
C8—C7—C12	118.1 (2)	C25—C24—C5	120.39 (17)
C8—C7—C1	119.70 (19)	C29—C24—C5	121.38 (15)
C12—C7—C1	122.07 (17)	C24—C25—C26	120.5 (3)
C7—C8—C9	120.4 (3)	C24—C25—H25	119.8
C7—C8—H8	119.8	C26—C25—H25	119.8
C9—C8—H8	119.8	C27—C26—C25	120.1 (3)
C10—C9—C8	121.0 (3)	C27—C26—H26	119.9
C10—C9—H9	119.5	C25—C26—H26	119.9
C8—C9—H9	119.5	C28—C27—C26	120.0 (2)
C9—C10—C11	119.6 (3)	C28—C27—H27	120.0
C9—C10—H10	120.2	C26—C27—H27	120.0
C11—C10—H10	120.2	C27—C28—C29	120.4 (3)
C10—C11—C12	120.0 (3)	C27—C28—H28	119.8
C10—C11—H11	120.0	C29—C28—H28	119.8
C12—C11—H11	120.0	C28—C29—C24	120.7 (2)
C7—C12—C11	120.9 (2)	C28—C29—H29	119.6
C7—C12—H12	119.6	C24—C29—H29	119.6
C11—C12—H12	119.6		
			
C6—N1—C1—C7	−68.92 (17)	C9—C10—C11—C12	−0.2 (4)
C5—N1—C1—C7	168.96 (12)	C8—C7—C12—C11	−0.1 (3)
C6—N1—C1—C2	170.20 (14)	C1—C7—C12—C11	−175.15 (19)
C5—N1—C1—C2	48.08 (18)	C10—C11—C12—C7	0.7 (4)
N1—C1—C2—C3	−51.25 (19)	C3—O1—C13—O2	2.1 (2)
C7—C1—C2—C3	−174.15 (14)	C3—O1—C13—C14	−179.60 (13)
C13—O1—C3—C2	102.68 (16)	O2—C13—C14—C15	173.29 (19)
C13—O1—C3—C4	−135.44 (14)	O1—C13—C14—C15	−5.0 (2)
C1—C2—C3—O1	−179.58 (12)	O2—C13—C14—C16	−5.9 (2)
C1—C2—C3—C4	60.16 (17)	O1—C13—C14—C16	175.90 (13)
O1—C3—C4—C22	62.12 (16)	C15—C14—C16—C21	−50.8 (3)
C2—C3—C4—C22	−178.14 (13)	C13—C14—C16—C21	128.38 (19)
O1—C3—C4—C23	−58.19 (16)	C15—C14—C16—C17	127.2 (2)
C2—C3—C4—C23	61.55 (16)	C13—C14—C16—C17	−53.7 (2)
O1—C3—C4—C5	179.71 (11)	C21—C16—C17—C18	0.5 (3)
C2—C3—C4—C5	−60.55 (15)	C14—C16—C17—C18	−177.43 (18)
C1—N1—C5—C24	−177.09 (12)	C16—C17—C18—C19	−0.4 (4)
C6—N1—C5—C24	61.42 (16)	C17—C18—C19—C20	−0.4 (4)
C1—N1—C5—C4	−52.00 (17)	C18—C19—C20—C21	1.0 (5)
C6—N1—C5—C4	−173.50 (13)	C17—C16—C21—C20	0.1 (3)
C3—C4—C5—N1	55.93 (15)	C14—C16—C21—C20	178.1 (2)
C22—C4—C5—N1	172.60 (13)	C19—C20—C21—C16	−0.9 (4)
C23—C4—C5—N1	−65.83 (17)	N1—C5—C24—C25	−142.18 (15)
C3—C4—C5—C24	−179.80 (12)	C4—C5—C24—C25	91.57 (18)
C22—C4—C5—C24	−63.13 (17)	N1—C5—C24—C29	39.07 (19)
C23—C4—C5—C24	58.43 (18)	C4—C5—C24—C29	−87.18 (17)
N1—C1—C7—C8	153.59 (17)	C29—C24—C25—C26	2.1 (3)
C2—C1—C7—C8	−82.9 (2)	C5—C24—C25—C26	−176.69 (18)
N1—C1—C7—C12	−31.4 (2)	C24—C25—C26—C27	−1.8 (3)
C2—C1—C7—C12	92.05 (19)	C25—C26—C27—C28	0.2 (4)
C12—C7—C8—C9	−1.0 (3)	C26—C27—C28—C29	1.0 (4)
C1—C7—C8—C9	174.1 (2)	C27—C28—C29—C24	−0.7 (3)
C7—C8—C9—C10	1.6 (4)	C25—C24—C29—C28	−0.9 (3)
C8—C9—C10—C11	−0.9 (5)	C5—C24—C29—C28	177.91 (17)

### 1,3,3-Trimethyl-2,6-diphenylpiperidin-4-yl 2-phenylprop-2-enoate Hydrogen-bond geometry (Å, º)

*Cg* is the centroid of the C16–C21 phenyl ring.

**Table:**

*D*—H···*A*	*D*—H	H···*A*	*D*···*A*	*D*—H···*A*
C12—H12···N1	0.93	2.56	2.869 (2)	100
C3—H3···O2	0.98	2.26	2.677 (2)	104
C15—H15*B*···O1	0.93	2.34	2.683 (2)	101
C22—H22*B*···O1	0.96	2.53	2.878 (2)	102
C23—H23*C*···O1	0.96	2.57	2.903 (2)	100
C18—H18···O2^i^	0.93	2.55	3.473 (3)	170
C15—H15*A*···*Cg*^ii^	0.93	2.94	3.613 (2)	130

Symmetry codes: (i) −*x*, −*y*−1, −*z*; (ii) *x*, *y*+1, *z*.
